# Alleged Death from Inhalation of Nitrous Oxyd Gas

**Published:** 1864-01

**Authors:** 


ALLEGED DEATH FROM INHALATION OF NITROUS OXYD GAS.—It was reported in the New York newspapers a few days ago, that a merchant of that city had died from the inhalation of nitrous oxyd gas. He had gone to the office of Dr. Joseph Burnett for the purpose of having a tooth extracted, who, at his request, administered nitrous oxyd gas as an anæsthetic. For some time after the tooth was extracted he appeared to recover from the effects of the anæsthetic. Soon, however, he complained of being ill, and grew worse so rapidly that a physician was called in, who administered the usual restoratives in such cases. He continued to grow worse, and died in about two hours after the tooth was extracted.

Dr. Geo. B. Bouton made a post-mortem examination on the body, when he found that deceased was very consumptive,—that one of his lungs was nearly gone, and the other considerably affected by disease. Dr. Bouton, in his testimony before the jury, gave it as his opinion, that if deceased had been in ordinary health, he would not have been injuriously affected by the inhalation of the gas.

The jury rendered the following verdict:

"We find that the deceased came to his death from congestion of the lungs induced by the administration of nitrous oxyd gas, for the purpose of extracting a tooth. We further state that we would exonerate the person who administered the gas, from all criminal intent; but we think there should be an examination made by competent persons, in all cases where it is contemplated to administer said gas."

It is a very common, though a very grave error to look upon every *post hoc* as a *propter hoc.* In view of the pathological condition of the lungs of the patient, we have little doubt that the same result would have followed the extraction of the tooth if no anæsthetic had been taken. When a person has so slight a hold on life as this man had, so insignificant a circumstance as the extraction of a tooth might sufficiently derange the nervous and circulatory systems as to induce the congestion that caused the death. Many a death, we have no doubt, is attributed to anæsthetics that is due to the disturbing causes alluded to.—*Med. and Surg. Reporter.*

